# Dynamic Changes of the Bone Marrow Niche: Mesenchymal Stromal Cells and Their Progeny During Aging and Leukemia

**DOI:** 10.3389/fcell.2021.714716

**Published:** 2021-08-10

**Authors:** Kevin Woods, Borhane Guezguez

**Affiliations:** ^1^German Consortium for Translational Cancer Research (DKTK), Heidelberg, Germany; ^2^Department of Hematology and Oncology, University Medical Center Mainz, Mainz, Germany; ^3^German Cancer Research Center (DKFZ), Heidelberg, Germany

**Keywords:** mesenchymal stromal cells, bone marrow niche, aging, leukemia, adipocyte, osteoblast

## Abstract

Mesenchymal stromal cells (MSCs) are a heterogenous cell population found in a wide range of tissues in the body, known for their nutrient-producing and immunomodulatory functions. In the bone marrow (BM), these MSCs are critical for the regulation of hematopoietic stem cells (HSC) that are responsible for daily blood production and functional immunity throughout an entire organism’s lifespan. Alongside other stromal cells, MSCs form a specialized microenvironment BM tissue called “niche” that tightly controls HSC self-renewal and differentiation. In addition, MSCs are crucial players in maintaining bone integrity and supply of hormonal nutrients due to their capacity to differentiate into osteoblasts and adipocytes which also contribute to cellular composition of the BM niche. However, MSCs are known to encompass a large heterogenous cell population that remains elusive and poorly defined. In this review, we focus on deciphering the BM-MSC biology through recent advances in single-cell identification of hierarchical subsets with distinct functionalities and transcriptional profiles. We also discuss the contribution of MSCs and their osteo-adipo progeny in modulating the complex direct cell-to-cell or indirect soluble factors-mediated interactions of the BM HSC niche during homeostasis, aging and myeloid malignancies. Lastly, we examine the therapeutic potential of MSCs for rejuvenation and anti-tumor remedy in clinical settings.

## Introduction

**FIGURE 1:**
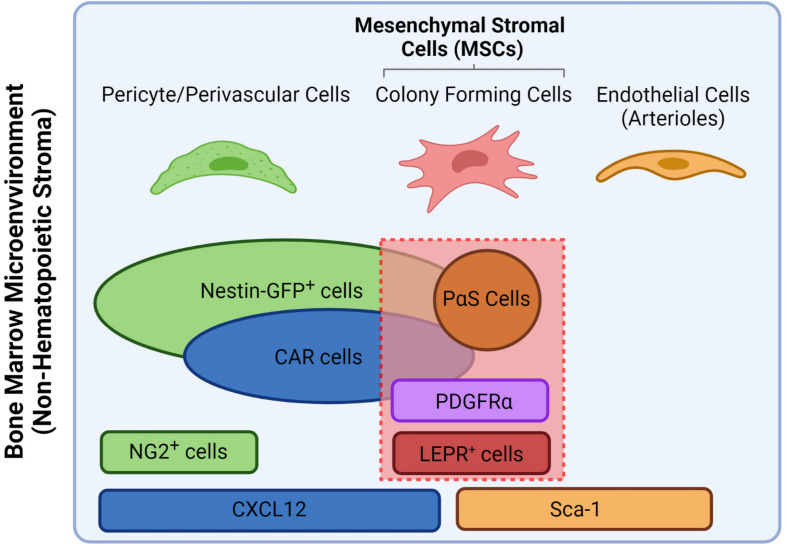
Nomenclature overview of different stromal populations including putative and gene markers and how they relate to MSCs. For the scope of this review, MSCs are defined as all colony forming cells that express both PDGFR-α and LEPR (Matsuzaki et al., 2014). PαS stand for PDGFR-α^+^/Sca-1^+^. Figure was generated using Biorender.com.

Located within specific anatomical zones of the skeleton, the bone marrow (BM) is a specialized microenvironment or “niche” that lodges cells of hematopoietic and mesenchymal origins in various hierarchical committed states. The main role of the BM niche is the tight control of cell-fate decisions of the hematopoietic stem cells (HSCs) and their progeny to sustain the daily supply in functional blood and immune cells throughout life. These environmental cues are produced by a variety of stromal cells that constitute the BM niche which mainly include neurons, endothelial cells and mesenchymal stromal cells (MSCs) (Pinho and Frenette, 2019). The latter are considered a versatile stem cell population due to their capacity to differentiate into bone (osteoblasts), cartilage (chondrocytes) and fat cells (adipocytes), thus playing a central role in HSCs maintenance, BM niche composition and life-long turnover and bone growth (Bianco and Robey, 2015). Due to their fibroblastic nature and heterogenous origin, MSCs have been referred to in the literature under different names which were accounted for in this review. In addition, prominent gene reporter-mouse models that helped investigate the role of stromal populations in the BM led to synonymous use of the reporter strains themselves as putative markers for MSC populations, which are different from their human counterparts (see Table 1). However, current consensus divided MSCs into subgroups based on their anatomical location which influence both their functional and phenotypic potentialities. Therefore, within the scope of this review, we refer to the nomenclature proposed by Matsuzaki et al. (2014) and revised by Ambrosi et al. (2019); according to which MSCs are defined as bone marrow stromal cells bearing trilineage potential and expressing both Leptin receptor (LEPR) and PDGF-receptor α (PDGFR-α) in human and mouse (see Figure 1). Acknowledging the presence of further heterogeneity within the MSCs compartment, we will review major niche factors contributed by the MSCs and their osteo-adipo progeny in sustaining hematopoiesis. We will also present the most recent advances in identifying MSCs subset heterogeneity and cellular hierarchy by single cell technologies and their impact on remodeling the BM during aging and myeloid leukemias. Consequently, we will highlight possible therapeutic options in targeting MSCs in clinical settings.

**TABLE 1:** Nomenclature of stromal populations based on genetic/putative markers.

Name	Used in	Refers to	Additional info	Organism
CAR-cell	Sugiyama et al., 2006	Endosteal niche, near HSC, CXCL12-expressing cells	Not the same as PDGFR-α ^+^/Sca1^+^ cells, but both have trilineage potential (Helbling et al., 2019)	Mouse, Human (Aoki et al., 2021)
LEPR-MSC	Ding et al., 2012	Scf-GFP expressing perivascular stromal cells	Express PDGF-R, CXCL12, not Nestin, perivascular niche	Mouse, Human
Mesenchymal stem cell	Jessop et al., 1994	Stem cell with multilineage potential		Mouse, Rat, Rabbit, Lamb, Human
Multipotent Mesenchymal stromal cell	Dominici et al., 2006	CD105^+^, CD73^+^, CD90^+^, CD45^–^, CD34^–^, CD11b^–^, CD79a^–^, CD19^–^, HLA-DR^–^	ISCT criteria	Human
Nestin^+^ MSC	Méndez-Ferrer et al., 2010	Mesenchymal stem cells	Mouse model for MSC	Mouse, Human (Pinho et al., 2013)
NG2^+^ pericyte	Kunisaki et al., 2013	Pericytes that control HSC quiescence, different from LEPR^+^ (sinusoidal) cells	Mouse model for MSC, also show trilineage potential	Mouse, Human (Kozanoglu et al., 2009)
PDGFR-α^+^-Sca1^+^ MSC (PαS)	Morikawa et al., 2009	Perivascular mesenchymal stromal cells		Mouse
Skeletal stem cell	Abdallah et al., 2004; Bianco and Robey, 2015	Mesenchymal stem cells		Human

## Functional MSC Heterogeneity: Location and Progeny Matters

**FIGURE 2:**
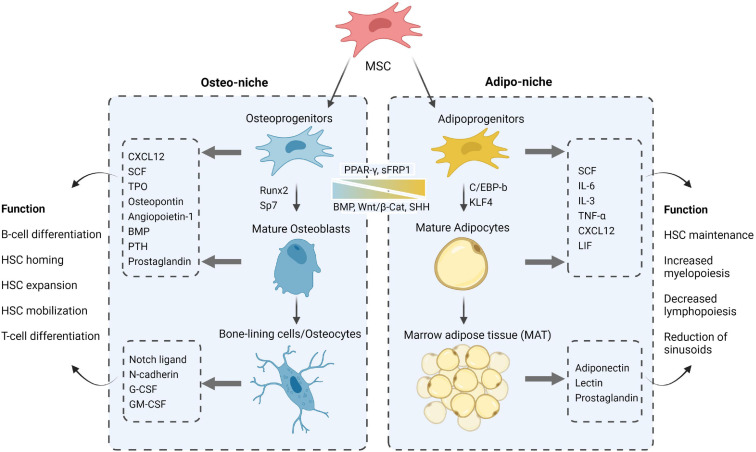
A closer look at the MSC progeny, their constituting roles via secreted factors and differentiation cues toward adipo- and osteogenesis in the respective niches. Figure was generated using Biorender.com.

The BM niche can be divided into two distinct regions based on the location of the cells, vascular flow and oxygen conditions they are exposed to which consequently define functional differences between MSCs within these distinct niche sites (see Figure 2):

*The endosteal bone marrow niche* represents 10% of total BM volume and comprises the MSCs with high osteolineage capacity including osteoprogenitors, osteoblasts, and osteocytes, which populate the inner surface of the bone along small arterioles and capillary vessels (Méndez-Ferrer et al., 2020). NG2^+^ pericytes and MSCs along with their osteo-progeny were shown to promote HSC quiescence through secretion of pro-survival and homing factors such as C-X-C Motif Chemokine Ligand 12 (CXCL12) (Wei and Frenette, 2018), Angiopoietin-1 (Ang-1) (Arai et al., 2004), thrombopoietin (TPO) (Yoshihara et al., 2007), and Notch ligands (Calvi et al., 2003; Guezguez et al., 2013); thereby reinforcing their tight contact with osteoblasts and maintaining the HSCs in a long-term non-cycling status (Qian et al., 2007; Loeffler and Schroeder, 2021). In accordance, the osteocalcin^+^ osteoblasts have been identified as a supportive “layer” niche due to their organization in follicle-like structures which surround HSCs and bind to them via N-cadherin- and Notch/Jagged1 mediated cell-cell interactions (Calvi et al., 2003; Zhang et al., 2003; Lawal et al., 2017). More recent reports indicate that the regulation of hematopoiesis by the osteolineage may also depend on it differentiation state (Sacchetti et al., 2007; Méndez-Ferrer et al., 2010; Calvi et al., 2012; He et al., 2017), as well as the close spatial localization of HSCs with the bone-lining cells of the endosteal niche (Lo Celso et al., 2009; Xie et al., 2009; Guezguez et al., 2013; Kim et al., 2017). These physical osteoblastic niche interactions controlling HSC fate are extensively influenced by a profusion of autocrine, paracrine, and endocrine factors such as bone morphogenetic proteins (Jung et al., 2008; Goldman et al., 2009; Khurana et al., 2014; Guo et al., 2018), growth factors (Yoon et al., 2012, 2017; Caselli et al., 2013), prostaglandins (Frisch et al., 2009; Hoggatt et al., 2009, 2013), shared cytokines/chemokines (Sugiyama et al., 2006; Ding and Morrison, 2013; Brylka and Schinke, 2019) and hormones such as the parathyroid hormone (PTH) (Calvi et al., 2001, 2003; Kuznetsov et al., 2004; Li et al., 2012). Although all of these molecules appear to be essential cornerstones for the preservation of bone microarchitecture and stem/progenitor cell homeostatic features within the BM, PTH has been identified as a key osteo-niche element linking MSCs and HSCs activities functionally and spatially (Adams et al., 2007; Li et al., 2012; Yu et al., 2012; Yao et al., 2014; Wein and Kronenberg, 2018). Additionally, osteoprogenitors were shown to be indispensable for B-cell differentiation by the release of Interleukin-7 (IL-7) and Insulin Growth Factor (IGF-1) which are critical for the maturation steps of B-cell progenitors (Wu et al., 2008; Yu et al., 2016). On the other hand, osteocytes were shown to restrict myelopoiesis by secreting granulocyte colony-stimulating factor (G-CSF) an important factor in HSC mobilization (Fulzele et al., 2013). The interdependence of endosteal BM niche inhabitants and the multifaceted signaling of MSCs and their osteo-lineage progeny in controlling HSC functions continue to be the object of intense investigation.

*The central/perivascular bone marrow niche* delineates 90% of total BM volume and englobes most of the vasculature that is enveloped with a variety of cells, including MSCs, pericytes, neurons along with adipocytes, which populate the central region of the bone shaft (Méndez-Ferrer et al., 2020). The BM vasculature in this region is enriched with arterioles that branch with thin-walled and fenestrated blood vessels called sinusoids. This endothelial architecture allows for the tight balance in the retention and activation of HSCs as well as the trafficking of their progenitors and mature immune cells back and forth the BM (Itkin et al., 2016). Along secretion of CXCL12, the LEPR^+^-MSCs enveloping the sinusoids are shown to produce stem cell factor (SCF, also known as KITL) that is required for long-term preservation of HSCs in the BM (Ding et al., 2012). Adipocytes, known to be a rich source in nutrients for the BM, also produce a variety of cytokines and factors involved in HSCs maintenance (SCF, IL-3, IL-6, CXCL12) (Kumar and Geiger, 2017) as well as inhibitors of hematopoiesis such as TGF-β1, a mediator of cell-cycle arrest (Scandura et al., 2004; Brenet et al., 2013) and lipocalin 2 (LCN2) that inhibits erythroid differentiation (Miharada et al., 2008). More intriguingly, accumulation of adipocytes as marrow adipose tissue (MAT) was also shown to reduce blood flow and suppress hematopoiesis through reduction of sinusoid caliber and microvasculature pruning (Scheller et al., 2016).

Overall, accumulating evidence has demonstrated a balance of MSCs differentiation commitment between osteoblastic and adipocytic lineages; as well as mutual dependency to ensure homeostasis that can be derailed during aging, chronic stress or cancer (Rendina-Ruedy and Rosen, 2017). However, possible feedback signals between osteo-adipo lineage and their parental MSCs as well as their impact on BM niche biology remains to be elucidated.

## Single-Cell MSC Heterogeneity: Lesson From Single Cell RNA Sequencing

### MSC Heterogeneity in the Murine Bone Marrow

With the advance of single-cell RNA sequencing technologies (scRNA-seq), traditionally homogenous cell populations reveal functionally different subclasses. The same is true for MSCs; recent well-designed scRNA-seq studies from different stromal gene-reporter mice shed some light on the murine bone marrow and help us to identify subclasses of MSCs. However, results from these studies varied greatly in number of identified MSC subsets due to different methods of BM extraction, cell sorting and sequencing depth (see Table 2). In summary, both “adipogenic” and “osteogenic” clusters can be identified regardless of the gene-reporter or surface MSC marker (LEPR^+^, CD51-/Sca1^+^, PDGFR- α^+^, Col2^+^) (Tikhonova et al., 2019; Wolock et al., 2019; Baccin et al., 2020; Zhong et al., 2020). Depending on gene set signatures, MSC can be subdivided into subsets with less differentiated and more stem-like features that are defined as mesenchymal progenitors or mesenchymal stem cells (Tikhonova et al., 2019; Zhong et al., 2020). Additionally, some of these studies also discerned “intermediate” MSC populations, suggesting that adipogenic and osteogenic differentiation is a continuous process with little definite cell states in-between (Tikhonova et al., 2019; Wolock et al., 2019; Leimkühler et al., 2021), as shown recently for the HSC compartment (Liggett and Sankaran, 2020).

**TABLE 2:** Comparison of recent sc-RNA seq experiments on murine bone marrow stroma.

Tissue obtained	Sorted on	Single cell method	Stromal population	Subclasses	Signature genes	Number of cells	References
BM flushed, bones crushed and digested with STEMxyme1, Dispase II, ACK lysis	CD71^–^/CD45^–^/CD3^–^/B220^–^/CD19^–^/Gr-1^–^/CD11b^–^	Chromium single cell 3′ Reagent V2 (10x genomics), Chromium Controller (10x Genomics)	LEPR^+^	N/A	LEPR^*hi*^, CXCL12^*me*^, KitL^*hi*^, Grem1^*hi*^, Angpt1^*me*^	20.896	Baryawno et al., 2019
				N/A	LEPR^*hi*^, CXCL12^*hi*^, KitL^*hi*^, Grem1^*lo*^, Angpt1^*me*^		
				N/A	LEPR^*hi*^, CXCL12^*hi*^, KitL^*hi*^, Grem1^*me*^, Angpt1^*hi*^		
				osteolineage	LEPRme, CXCL12^*me*^, KitL^*me*^, Grem1^*me*^, Angpt1^*me*^		
BM flushed, bones digested with Liberase^TM^ and DNAseI	Lepr-tdT^+^	Chromium single cell 3′ Reagent V2 (10x genomics), Chromium Controller (10x Genomics)	LEPR^+^	Adipogenic (Mgp^*hi*^)	Mgp, Gpx3, Serping1, Lepr, Tmem176b, Igfbp5, Malat1, C1ra, C4b, Epas1	17.374	Tikhonova et al., 2019
				Adipogenic (LPL^*hi*^)	Lpl, Scp2, Fstl1, Rgcc, Mrps6, Pdzrn4, Mmd, Npc2, Slc5a3, Angpt1		
				Osteo-primed (Wif1^*hi*^)	Col8a1, Kcnk2, Ndnf, 150015O10Rik, Palld, Tnfrsf19, Cldn10, Slc20a2, Limch1, Fhl2		
				Osteo-primed (Spp1^*hi*^)	Col1a1, Spp1, Col13a1, Mmp13, Ifitm5, Serpine2, Mef2c, Ibsp, Itgb5, Aqp1		
Bones crushed and flushed, fragments digested with collagenase/dispase	CD45^–^/Ter119 ^–^/CD31^–^	inDrops (Weitz et al., 2015)	CD51^–^ Sca1^+^	(Pre)-Adipocyte/Adipocyte progenitor	Nr4a1, CXCL1, Ifrd1, Fosb, Ccl2, LEPR, Kitl, Adipoq	2.847	Wolock et al., 2019
				MSC	Cbln1, Clec2d, Pdzrn4, Cybb, Rspo2, LEPR, CXCL12, Kitl, Adipoq		
				Osteoblast/chondrocyte progenitor	Postn, Wif1, Mmp9, Kcnk2, Limch1, LEPR, CXCL12, Kitl, Adipoq, Alpl, Col1a1, MMP13, Spp1		
				Pre-osteoblast/chondrocyte	Postn, Wif1, Mmp9, Kcnk2, Limch1, Alpl, Sp7, Col1a1, Mmp13, Spp1		
				Pro-osteoblast	Col1a1, Bglap, Col11a2, Col11a1, Bglap2, Alpl, Sp7, Col1a1		
				Pro-chondrocyte	Dmp1, Ackr3, Spp1, Ank, CD44, Col1a1, Mmp13, Mepe, Spp1		
Bones crushed, cells filtered, MACS separation (CD5^–^, CD45R^–^, CD11b^–^, Ly-6G/C^–^, 7-4^–^, Ter-119^–^	CD41^–^, CD3^–^, CD11b^–^, Gr1^–^, Ter119^–^, CD45R^–^, CD45.1^–^, CD45.2^–^, Sca1^–^, CD31^–^, CD51^–^	Chromium single cell 3′ Reagent V2 (10x genomics),	LEPR^+^, PDGF-R-α^+^, Vcam1^+^, CXCL12^+^, Kitl^+^, Angpt1^+^	Adipogenic	Mgp, Adipoq, CXCL12, Kitl	2.294	Leimkühler et al., 2021
				Osteogenic	Spp1, WIf1, Ibsp, Sp7, Bglap		
				Transitioning	Chromatin remodeling, RNA processing (Top GO-terms, no gene list stated)		
				Interferon-responsive	Chromatin remodeling, RNA processing (Top GO-terms, no gene list stated)		
Bones crushed, bone chips digested with Collagenase II/Dispase, filtered, ACK lysis, lineage depletion (Dynabeads)	Ter119^–^, CD41^–^, CD45^–^, CD51^–^, CD71^–^, VCAM1^+^, CD200^–^, CD61^–^	Chromium single cell 3′ Reagent V2 (10x genomics)	PDGF-R-α^+^	Adipo-CAR	Cxcl12, Tmem176b, Hp, Lpl, Tmem176a, H2-D1, Apoe, Gas6, Adipoq, Esm1	7.497	Baccin et al., 2020
				Osteo-CAR	Tnc, Igfbp4, Wif1, Cd63, Cxcl12, Olfml3, H2-D1, Kcnk2, Gas6, Serpine2		
				NG2^+^	Cd63, Spp1, Serpine2, Tnc, Mmp13, Ibsp, Cfh, Timp1, Cd200, Serpinh1		
Bones scraped to remove periosteum, bones flushed, bone chips digested with proteases	Col2-Td^+^	Chromium Controller V3 (10x genomics)	Col2	Early mesenchymal progenitors	Ly6a, CD34, Thy1, Mfap5, Gsn, Clec3b	7.585	Zhong et al., 2020
				Late mesenchymal progenitors	Aspn, Edil3, Tnn, Postn, Ostn, Dkk3		
				Osteoblasts/Osteocytes	Sp7, Runx2, Col1a1, Ibsp, Bglap2, DMP1		
				Adipocytes	Cebpa, Cebpb, PParg, Lpl, Adipoq, Apoe		
				Chondrocytes	Sox9, Col2a1, Col10a1, Pth1r, Acan, Ihh		

### MSC Heterogeneity in the Human Bone Marrow

There are few comparable scRNA-seq studies of the MSC heterogeneity in human. This is in parts due to the scarcity of material and the difficulties in getting consistent cell content from BM aspirates. Compared to full mouse bones, human BM aspirates contain very few MSCs within the range of 0.001–0.01% of total cellularity (Pittenger et al., 1999; Qin et al., 2021). In addition, the donors’ age and sex also influences MSCs phenotype and content (Siegel et al., 2013), adding another layer of heterogeneity to the analyzed samples. Further approaches to increase MSCs content from human material require enrichment applications by cell sorting strategies and *in-vitro* expansion, inevitably leading to a loss of subpopulations and altered gene expression while affecting resolution capacity of scRNA-seq (Ghazanfari et al., 2017; Liu et al., 2019). The current high cost of single cell-sequencing and the low MSCs content typically result in scRNA-seq experiments with fewer than 100 MSCs, resulting in difficulty for sub-clustering analysis. In consequence, these experiments translate BM-derived MSCs as a single “homogenous” population that is compared to other MSC sources (Barrett et al., 2019; Zhou et al., 2019). In a recent scRNA-seq mapping experiment of large BM hematopoietic cell populations, a small amount of heterogeneous MSCs were captured, with one subset expressing high levels of the key bone marrow-homing cytokine CXCL12. This MSC subclass was later validated by high enrichment of CXCL12 and other key MSC signature genes from FACS-based isolation of CD13^+^CD11a− cells (Triana et al., 2021). Another notable exception is a study done by Wang et al. (2020), where a total of 14.494 CD271^+^ BM-MNCs were analyzed. This study led to similar findings compared to the murine experiments, revealing adipo-, osteo-, and chondrogenic clusters as well as two terminal clusters that could represent senescent cells (see Table 3).

**TABLE 3:** Human MSCs subsets based on sc-RNA sequencing of human BM tissue.

Tissue obtained	Sorted on	Single cell method	Stromal population	Subclasses	Signature genes	References
Bone marrow aspirate, density gradient (Ficoll 1.077), lysis, CD271^+^ MACS separation (Miltenyi)	No sorting	Chromium single cell 3′ Reagent V2 (10x genomics)	LEPR^+^	Osteogenic	XIST, COL6A3, COL1A1, VCAN, C7, THY1, ADM, ANGPTL4, PGF, COL6A2	Wang et al., 2020
				Adipogenic	HP, IGHG3, IGKC, FBLN1, RETREG1, APOD, CTGF, ADIPOQ, MGP, RPS26	
				Terminal 1	FTL, RPS12, RPL30, RPS3A, RPL10, RPL34, TPT1, RPL12, RPS4X, RPS24	
				Terminal 2	XIST, MALAT1, CSAD, NKTR, KCNQ1OT1, FUS, GOLGB1, WSB1, CCNL2, CCNL1	
				Chondrogenic	S100A8, S100A9, S100A12, CAMP, LTF, MNDA, S100A4, MMP9, LCN2, LYZ	

**FIGURE 3:**
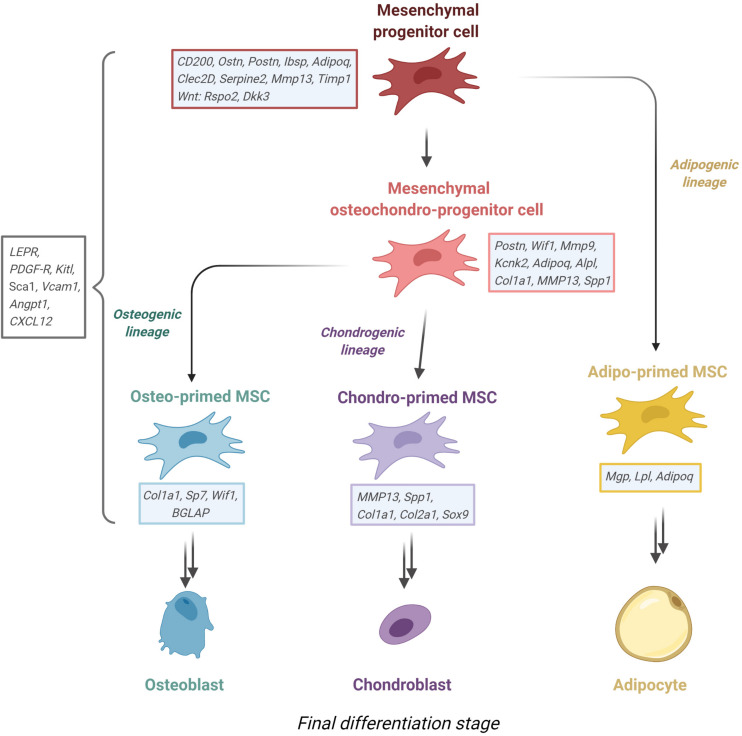
Proposed MSC lineage tree derived from recent sc-RNA sequencing experiments from murine stroma. Mesenchymal progenitor cells give rise to either adipo-primed MSC or an osteochondro-progenitor, which in turn gives rise to osteo-primed or chondro-primed MSC. Noted beside each entity are the most defining upregulated genes. Figure was generated using Biorender.com.

Recent advances in species transfer learning methods allowed the harmonization of single cell-sequencing data from mouse to human, finding equivalent clusters of cells in BM of both species (Stumpf et al., 2020). While this approach is useful to generalize findings across species, it is also limited in several ways, e.g., only orthologous genes are transferred. Even within the same cluster of cells of each respective species, there are significant transcriptional profile differences, for instance in GO terms (Wang et al., 2020), posing the question whether these cells truly play the same role in mouse and man. With all these factors in mind, we propose the following hierarchy of the MSCs and their progeny in the BM that is validated in both mouse and human (see Figure 3), with the outlook that future studies will reconcile the missing phylogenic gaps for a unified cellular portrait of MSCs.

## MSC Changes in Aging Bone Marrow

During aging, the BM undergoes drastic changes with loss in osteoblasts and increase in adipocytes content leading to a change in overall cellularity, bone density and a shift in anatomical distribution from “red” to “yellow” marrow (reviewed in detail by Goltzman, 2019). In recent years, focus has been set on MSCs as the main source of these changes with the hope of ameliorating age-related alterations such as osteoporosis. In accordance with age-shift toward an adipogenic phenotype, recent scRNA-seq studies in old mice found that MSC subsets with adipogenic potential (AdipoCAR) increase excessively alongside with a depletion of mature osteoblasts (Zhong et al., 2020; Dolgalev and Tikhonova, 2021). However, there are conflicting reports about the overall number of MSCs during BM aging, with some studies indicating no changes (Aguilar-Navarro et al., 2020; Meza-León et al., 2021) while a majority of reports indicates an increase in some subsets of MSCs (Maryanovich et al., 2018; Frisch et al., 2019; Singh et al., 2019). These discrepancies can be explained due to different methodological approaches and is further underlined by pathological observations demonstrating divergent cellular BM changes between mouse and human during aging (Meza-León et al., 2021). However, common mammalian features of functional deregulation have been described in deciphering the age-related changes of MSCs:

### Direct Deregulation

The observed hypocellularity in aged individuals can be attributed to altered MSCs differentiation capacity toward expansion of adipocytes and increased risk of osteoporosis. Indeed, MSC show an age-dependent lineage switch between the osteogenic and adipogenic fate. Under normal conditions, MSCs homeostasis is regulated by transcription factors PPARγ and C/EBPs toward the adipogenic lineage and Runx2 and Osterix for the osteogenic lineage. These in turn are controlled by cell adhesion toward extracellular matrix (ECM)-Integrins and molecular signaling from Wnt, Notch, BMP, Hedgehog and FGF pathways (Figure 2 and reviewed in detail by Chen et al., 2016). In consequence, these pathways are of special interest to identify aging effects. Clinical data demonstrated that patients with osteoporosis or age-dependent bone loss display low activity of Wnt/β-Catenin signaling in MSC while RhoA-Rock activity is inversely correlated with β-Catenin signaling in BM-MSCs from elderly human subjects (Stevens et al., 2010; Shi et al., 2021). The decrease of Wnt-signaling can be attributed in parts to a decrease in Yes-associated protein (YAP) in MSCs during aging, a co-transcription factor that was identified recently as an interaction partner of the β-Catenin complex (Pan et al., 2018). Recent studies revealed additional transcriptional regulatory mechanisms of the Wnt pathway by different classes of non-coding RNAs, such as microRNA miR-146a, whose levels increased in patients suffering from bone fragility (Saferding et al., 2020). Other circular (Ji et al., 2021) and long (Li et al., 2018) non-coding RNA were also found to play a role in lineage commitment by inhibiting the Runx2 transcriptional complex needed for osteoblastic differentiation. The delicate balance between osteo- and adipogenesis via the different transcriptional programs can also be influenced by Bmi1, a polycomb group protein that restricts adipogenic differentiation (Kato et al., 2019) and is downregulated in aged mice (Zheng et al., 2021). Similar to Wnt pathway, Indian Hedgehog-(IHH) signaling, which induces chondrogenesis in human MSCs (Steinert et al., 2012), was shown to be decreased in peroxide-induced senescent MSCs and MSCs from older donors (Al-Azab et al., 2020). Furthermore, adipogenesis and osteoclastogenesis is promoted indirectly by Sirtuin 3 (Sirt-3), a metabolic regulator of cellular senescence driven by the mTOR-pathway, that is found to be elevated in aged male mice and resulting in cortical bone loss (Ho et al., 2017).

### Senescence

Beside an apparent increase in MSCs content during aging, there is also a substantial increase in their senescence contributing to a decrease in the osteoblastic lineage and accelerated bone loss. A possible reason for this might be the development of aging-dependent inflammatory niche signaling, leading to noticeably increased IL-1α levels (which induces senescence via Bmi-1 downregulation) as well as IL-6 and TGF-β (Valletta et al., 2020; Zheng et al., 2021). A wide range of non-coding RNA have also been shown to regulate senescence both in mice and human (reviewed in Cai et al., 2021). In addition, aged MSCs produce high amounts of CXCL2 and CXCL5 chemokines, which contribute to the senescence-associated secretory phenotype (SASP) (Helbling et al., 2019). RANKL, an osteoclastogenic cytokine, has been shown to be increasingly secreted by MSCs in aged mice (Lin et al., 2017), leading to bone loss (Kim et al., 2020). Cellular senescence also leads to a decrease in Optineurin (OPTN), an autophagy receptor therefore contributing to osteoporosis alongside with accumulation of the OPTN substrate fatty acid binding protein 3 (FABP3) (Liu et al., 2020).

### Indirect Deregulation

A possible mechanism for the observed increase in MSCs might be driven by sensory adrenergic denervation that occurs in the aging microenvironment (Neuropathy), which in turn leads to reduced negative regulation of MSCs pool size and to the expansion of certain subsets holding adipogenic potential (Maryanovich et al., 2018; Ho et al., 2019). These shifts in BM content are further exacerbated by an increase in endothelial cell numbers and a regression of arteriolar structures (Kusumbe et al., 2014). Such BM stromal transformations increases the risk toward a myeloid-skewing differentiation of HSCs and can potentially lead to clonal hematopoiesis and subsequent hematological neoplasia (Steensma and Ebert, 2020).

**FIGURE 4:**
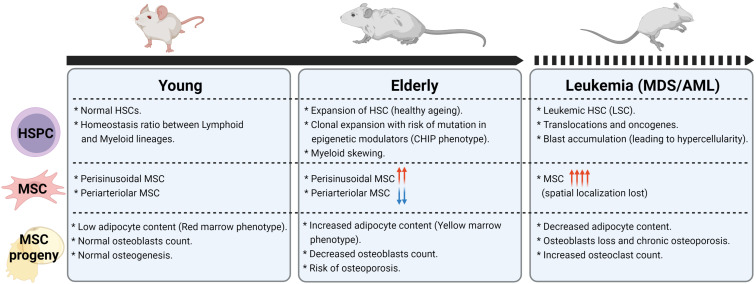
Changes in HSPC, MSC, and MSC progeny frequencies in aging and leukemia. Figure was generated using Biorender.com.

The aforementioned changes in the MSC niche are summarized in Figure 4.

### Addressing Age-Related Changes in the Niche

In recent years, focus on reverting cellular senescence became of major interest in addressing the aging-associated changes of MSCs. These approaches involve targeting the metabolic regulators Sirtuins 1 and 3 (Ma et al., 2017, 2020), pro-longevity growth factors such as fibroblast growth factor 21 (FGF-21) (Li et al., 2019) and downstream targets of HIF1α such as macrophage migration inhibitory factor (MIF) (Xia et al., 2015). A recent promising target is the hormone Lipocalin-2 (LCN2) that was previously shown to have a beneficial role in the regulation of various aspects of energy metabolism, especially in promoting fatty acid oxidation (Guo et al., 2010; Paton et al., 2013; Zhang et al., 2014). Further studies demonstrated that overexpression of LCN2 protect MSCs against stress-induced senescence and improve their paracrine and regenerative potentialities (Halabian et al., 2013; Bahmani et al., 2014). Furthermore, an LCN2 transgenic mouse model driven by bone-specific type 1 collagen, an osteolineage-specific promoter, showed expansion of long-term HSCs with higher clonogenic capacity due to elevated levels of CXCL12, SCF and matrix metalloproteinase inhibitors released by the BM niche (Costa et al., 2017). It has also been shown that osteoblasts, which decrease during aging, are the major source for blood circulating LCN2 in the body (Mosialou et al., 2020). Taken together, these findings suggest a beneficial effect of LCN2 supplementation on promoting hematopoieisis and stabilizing the aging BM microenvironment that would require further investigation for potential therapeutic applications.

In parallel, rewiring the MSC differentiation balance, originally explored as a rejuvenation strategy for treating osteoporosis, is currently under investigation as potential regenerative therapy to restore healthy hematopoiesis. One major example is the intermittent treatment with PTH or PTH-related peptide (PTHrP), shown to exert a well-known anabolic effect on the skeleton (Osagie-Clouard et al., 2017) and induction of HSC expansion (Calvi et al., 2003; Adams et al., 2007). Further studies demonstrated that Nestin^+^ MSCs isolated from PTH-treated mice displays enhanced proliferation and differentiation into osteoblasts in culture (Méndez-Ferrer et al., 2010; Ding et al., 2012); as well as increased osteogenic differentiation capacity *in vivo* (Fan et al., 2017). Other studies based on drug screening of natural senolytic substances such as Celastrol and Quercetin 3-O-β-D-galactopyranoside was also shown to promote osteogenesis and inhibit adipogenesis *in vitro* through PGC-1α signaling (Li et al., 2020; Oh et al., 2020). On a similar note, inhibition of the mTOR-pathway was shown to extensively prolong life-span in mice (Papadopoli et al., 2019), including revitalized pluripotency of human MSCs *in vitro* (Antonioli et al., 2019). Epigenetic modifiers were also recently proposed to revert the fat-bone-imbalance in skeletal aging, especially Lysine Demethylase 4B, which was shown to regulate β-catenin/Smad1 signaling toward MSC rejuvenation (Deng et al., 2021). Lastly, rejuvenated MSCs could also be interesting for *ex vivo* HSCs expansion in the context of stem cell transplantation therapies. As such, a recent and elegant co-culture study of HSCs with MSCs allowed to identify a set of “rejuvenating” transcription factors (Klf7, Ostf1, Xbp1, Irf3, and Irf7), that when over-expressed in MSC induces expansion of HSCs with enhanced regenerative and engraftment capacity while preventing accumulation of DNA damage (Nakahara et al., 2019).

In summary, most of these anti-aging approaches will require further validation prior possible translation toward clinical applications and other stromal targets not cited in this review are also currently under investigation (reviewed in more detail by Meng et al., 2020).

## MSC Heterogeneity in Myeloid Malignancies

Myeloid malignancies are clonal blood diseases arising from HSCs or subsequent progenitor cells that acquired oncogenic mutations and/or chromosomal translocations over a period of several years. Depending on the etiology of the disease, myeloid malignancies comprise chronic stages (including myelodysplastic syndromes: MDS, myeloproliferative neoplasms: MPN and chronic myelomonocytic leukemia: CMML) and acute stages encompassing different subtypes of Acute Myeloid Leukemia (AML) (Arber et al., 2016; Sperling et al., 2017; Vetrie et al., 2020; Witkowski et al., 2020). A large body of work demonstrated direct and indirect involvement of the BM niche in supporting neoplastic and leukemic cells during the development of myeloid malignancies. These tumorigenic features include advantageous release of pro-survival factors, competition in niche space with healthy HSCs, stromal reprogramming and physical protection against therapy (Méndez-Ferrer et al., 2020; Witkowski et al., 2020).

### MSC Niche-Driven Hematological Malignancies

Genetic mutation in mouse models affecting MSCs or their osteolineage progeny can induce different types of myeloid malignancies. For instance, activating-mutations in Nestin^+^ MSCs of the protein tyrosine phosphatase SHP2 (a positive regulator of the RAS signaling pathway) can lead to the development of childhood-like MPN by hyperactivating HSCs via overproduction of the CC-chemokine CCL3 and IL-1β (Dong et al., 2016). By contrast, deletion of the microRNA regulator DICER-1 in the Osterix^+^ osteolineage cells, prompt a pre-leukemia disease that mirrors human MDS and can evolve into secondary AML (Raaijmakers et al., 2010). Similarly, induction of Shwachman-Diamond syndrome mutation in Osterix^+^ stromal cells was shown to drive MDS evolution through the S100A8/9-TLR inflammatory signaling axis as a common driving mechanism of genotoxic stress that predicts AML progression in human patients (Zambetti et al., 2016). More recently, osteoblasts have also emerged as critical drivers of MDS via activating mutations in β-catenin signaling that can lead to progression to overt AML in mice (Kode et al., 2014; Stoddart et al., 2017). This aberrant activation of β-catenin signaling is also found in stromal cells of MDS patients along with DICER-1 dysregulation (Santamaría et al., 2012; Ozdogan et al., 2017) correlating with adverse prognosis (Bhagat et al., 2017).

### MSC Niche Reprogramming by Leukemia

Neoplastic and malignant cells can further remodel the MSC niche by specifically targeting the osteoblastic progeny during the stepwise disease progression from pre-leukemia stage (MDS/MPN) to overt AML (Yamaguchi et al., 2021). Specifically, it was shown that both MDS and MPN cells secrete inflammatory mediators such as CCL3 and TPO, thereby driving transformation of the MSC niche toward a highly supportive milieu for leukemic cell expansion at the expense of normal hematopoiesis (Schepers et al., 2013; Medyouf et al., 2014). This is consistent with xenograft studies suggesting that the MSC niche also provides a chemo-resistant niche for leukemic blasts (Ishikawa et al., 2007; Duan et al., 2014; Bertoli et al., 2018; Boutin et al., 2020).

Healthy Nestin^+^ MSCs and osteoblasts can also be indirect targets of sympathetic neuropathy (through β2-adrenergic signaling) in models of myeloid malignancies, leading either to aberrant expansion or loss of Nestin^+^ MSCs while restricting the numbers of mature osteoblasts in both MLL-AF9-AML (Hanoun et al., 2014) and JAK2V617F-MPN mouse models (Arranz et al., 2014). As a result, the impaired MSC niche promotes expansion of mutant HSCs and facilitates disease progression by loss of expression of HSC-retention factors, including CXCL12, SCF, ANG1, and VCAM1 (Arranz et al., 2014; Hanoun et al., 2014). Collectively, this is in agreement with clinical observations of stromal cells from MDS/AML patients, where expression of cell-surface molecules involved in interaction with HSCs is decreased (Geyh et al., 2013), whereas the population of human MSCs is increased, favoring blast expansion (Kim et al., 2015). In addition, osteogenic differentiation is significantly impaired by remodeling of the vasculature leading to reduced osteocalcin serum levels and deficiency in bone growth (Geyh et al., 2016; Duarte et al., 2018; Kumar et al., 2018), which is in line with reports of osteopenia or osteoporosis observed in newly diagnosed children or adults with acute Leukemia (Datzmann et al., 2018; Ruchlemer et al., 2018; Ahn and Suh, 2020).

### Mapping MSC Niche Heterogeneity in Leukemia

Despite the multiple functional studies investigating the role of the BM niche, little is known on the extent of transcriptional reshape of the MSC populations in myeloid malignancies, but recent scRNA-seq studies led to a better understanding of lineage shift and disease specificity. In AML context, single cell data revealed a concomitant decrease in committed osteolineage LepR^+^-MSCs in an MLL-AF9 mouse model along with an increase in pre-osteoblasts, suggesting a block in osteolineage maturation (Baryawno et al., 2019). This osteogenic differentiation blockade was further accompanied by a loss of transcriptional expression of multiple HSC niche factors (*Vcam-1, CXCL-12, SCF, Angpt, Il-7, CSF1*) and gene expression changes were also observed in endothelial cells and adipocytic populations (Baryawno et al., 2019). In a similar manner, RNA-seq studies on BM stroma from both mouse and human MPN shed light on the functional contributions of individual cellular components of the MSC population to myelofibrosis (Leimkühler et al., 2021). ScRNA-seq analysis demonstrated a fate switch between distinct precursor cells and MSC populations during stress-injury induced by malignant MPN clones. Two distinct MSC populations were shown to be the main drivers of BM fibrosis in mouse and human MPN. These two MSC populations are of LepR^+^ origin and are either adipogenic or osteogenic-biased progenitor populations. During MPN disease evolution, these MSC populations were demonstrated to be functionally reprogrammed into Collagen-producing myofibroblasts, reminiscent of Gli-1^+^ fibrosis-driving cells (Schneider et al., 2017) and leading to the excess deposition of ECM in BM which is considered one of the hallmarks of overt myelofibrosis (Barbui et al., 2018). Interestingly, all other MSC subsets were also shown to be reprogrammed into the production of non-collagenous ECM with scaffolding function for collagen fibrosis. This aberrant lineage shift was due to increased stromal expression of chronic inflammatory signals, especially TGF-β and S100A8/S100A9, leading toward a loss of hematopoiesis support (Vogl et al., 2018; Ribezzo et al., 2019).

Although more effort is necessary to unravel the MSC changes in different myeloid malignancies stages, all functional and genetic data indicate a shift toward an accumulation of MSC with adipogenic potential (Figure 4) that might be instrumental in disease evolution and should be explored further to specify therapeutic targeting.

### Development of MSC Therapies for Myeloid Malignancies

Given the central role of MSCs in the maintenance of both HSC and leukemic blasts, numerous studies investigated their potential direct therapeutic use in hematopoietic malignancies such as MDS and AML (reviewed in Fathi et al., 2019; Lee et al., 2019). Early co-culture studies of MSC and leukemia cells displayed contradictory results: either increased blast survival (Garrido et al., 2001) or anti-leukemic effects through the induction of apoptosis and cell cycle arrest (Liang et al., 2008; Tian et al., 2010). More broadly, a direct use of MSCs as a cellular anti-cancer therapy also proved to be difficult since the cells do not survive long enough to exhibit any beneficial effects (Levy et al., 2020) and were even shown to promote tumor growth in mouse models of MLL-AF9 AML and metastasic solid cancers (Okumura et al., 2009; Spaeth et al., 2009; Xu et al., 2009; Hanoun et al., 2014).

Acknowledging this functional duality of MSCs in leukemia growth, further research was directed in developing antibodies or compounds that target specifically the supportive malignant cues, more prominently toward the inhibition of the CXCL12-CXCR4 axis (Zhang et al., 2012; Kuhne et al., 2013) and IL6 signaling (Stevens et al., 2017). These promising compounds are currently being tested in combination with standard chemotherapy or allogenic transplantation settings in clinical trials of high-risk MDS and refractory AML patients (Martínez-Cuadrón et al., 2018; Roboz et al., 2018; Michelis et al., 2019; Bose et al., 2020). On the other hand, the anti-tumoral effects displayed by MSCs were attributed to small secreted factors (Maguire, 2013; Moll et al., 2020; Wu et al., 2020) and led to increased interest in the use of MSC secretome for anti-leukemic therapy as well as for a wide array of other diseases, such as ischemic, neuroinflammatory and pulmonary malignancies (reviewed in Harman et al., 2021). Collective proteomic studies demonstrated that the MSC secretome consists of trophic factors (e.g., FGF, HGF, VEGF), cytokines (e.g., IL-6, TGFβ-1…), hormones, small peptides (e.g., SCF, PTG, Leptin) and extracellular vesicles (EVs) containing miRNA, mRNA and biologically active proteins (Chulpanova et al., 2018). In consequence, cell-free therapy options are considered more promising for clinical applications (Hmadcha et al., 2020). However, it was shown that EVs from MSC can also contribute to tumor cell migration and growth by activation of Wnt, Erk or Akt pathways (Lin et al., 2013; Gu et al., 2016; Shi et al., 2016). EV content is dependent on many factors, such as MSC source (adipose tissue, umbilical cord, bone marrow), donor age, individual donor-specific influences, sampling method and other factors (Costa et al., 2021). This high variance in EV content hinders consistent therapeutic results, pushing the focus toward developing well-defined, standardized EVs (Lener et al., 2015); as well as engineering MSC-derived EVs that are loaded with anti-tumoral drugs or siRNA (Current clinical trials NCT03608631 and NCT01294072). Therefore, future studies are crucial to decipher the real potential of MSC-derived secretome and EVs for anti-leukemic therapies.

Using a holistic view, recent bioengineering advances were made in recreating *in situ* BM stroma through organ-on-a-chip devices that would allow to investigate MSC-mediated chemo-resistance mechanisms and assess therapy efficacy of new anti-tumor compounds (reviewed in Santos Rosalem et al., 2020). Similar approaches using biomimetic scaffolds capable of mimicking bone extracellular-matrix were also used to study MSC transcriptional and immunomodulatory alterations by MDS/AML blast cells (Abarrategi et al., 2017; Mian et al., 2021) and allowed recently for the discovery of a novel AML-MSCs selective CaV1.2 channel blocker drug, Lercanidipine, that is able to impair leukemia progression when administered *in vivo* (Borella et al., 2021). Collectively, although promising targets and drugs are currently further characterized toward translational applications, a careful development of MSC-based cell therapies will be primordial to boost ant-cancer properties while eliminating tumor-promoting effects.

## Concluding Remarks

MSCs represent a key component of the BM microenvironment, exerting multiple functions that are fundamental for tissue homeostasis, the support of the hematopoietic niche and the modulation of the immune system response during injury or infection. These activities are carried out through the secretion of a wide variety of factors, such as growth factors, cytokines and EVs. The aging process imposes profound modifications of both the morphology and functions of MSCs, leading to the development of a proinflammatory environment. Increasing evidence demonstrate that this reshape of the MSC niche is exacerbated during disease progression in hematologic malignancies by protecting cancer cells from apoptosis and inducing chemoresistance. Although our understanding of MSC niche contributions to aging and Leukemia has hugely increased over the last decades, more knowledge is required to harness the depth of complex MSC interactions with the highly polyclonal nature of aberrant HSCs or leukemic cells driving disease heterogeneity in MDS/AML. Moreover, many questions remain unresolved; in particular, whether the phenotypes and molecular mechanisms identified *in vitro* or in mouse models are maintained and therapeutically relevant in the human disease. In addition, the use of human leukemia samples in understanding aberrant MSC niche biology is currently hindered as clinical standard diagnoses are made on BM aspirates that disrupt BM architecture. Recent developments in single-cell sequencing and imaging technologies have made it possible to assess the heterogenous composition and diverse cellular and biochemical interactions present throughout complex tissue. Future integrative single-cell studies aimed at identifying the diverse network of cellular and biochemical interactions underlying the MSC niche may uncover unappreciated regulators or pathways controlling the BM aging process and cancer reprogramming and could lead to the development of novel therapeutic strategies aimed at improving health of the aging population or tackle chemoresistance in hematological malignancies.

## Author Contributions

BG and KW designed and edited the figures and tables. Both authors contributed to the manuscript.

## Conflict of Interest

The authors declare that the research was conducted in the absence of any commercial or financial relationships that could be construed as a potential conflict of interest.

## Publisher’s Note

All claims expressed in this article are solely those of the authors and do not necessarily represent those of their affiliated organizations, or those of the publisher, the editors and the reviewers. Any product that may be evaluated in this article, or claim that may be made by its manufacturer, is not guaranteed or endorsed by the publisher.

## Abbreviations

AML: Acute Myeloid Leukemia
B-ALL: B-cell Acute Lymphoblastic Leukemia
BM: Bone Marrow
CAR-cells: CXCL12-Abundant Reticular Cells
CML: Chronic Myeloid Leukemia
CMML: Chronic Myelomonocytic Leukemia
ECM: Extracellular Matrix
HSC: Hematopoietic Stem Cell
IHH: Indian Hedgehog
LEPR: Leptin Receptor
MDS: Myelodysplastic Syndrome
MF: Myelofibrosis
MIF: Macrophage Migration Inhibitory Factor
MNC: Mononuclear Cells
MPN: Myeloproliferative Neoplasm
MSC: Mesenchymal Stromal Cell
mTOR: mechanistic Target Of Rapamycin
NG2: Neural/glial antigen 2
PDGF-R: Platelet-Derived Growth Factor-Receptor
PGC-1 α: Peroxisome proliferator-activated receptor gamma coactivator 1-alpha
PTH: Parathyroid Hormone
P α S: PDGF-R-a^+^/Sca-1^+^
SASP: Senescence-Associated Secretory Phenotype
SCF: Stem Cell Factor
TPO: Thrombopoietin
YAP: Yes-Associated Protein.
